# Clinical and Biological Markers of Frailty Syndrome in Patients Undergoing Elective Percutaneous Coronary Intervention

**DOI:** 10.3390/diagnostics14232663

**Published:** 2024-11-26

**Authors:** Kristina Krivoshapova, Daria Tsygankova, Anastasiya Neeshpapa, Anastasia Kareeva, Alexandr Kokov, Evgeny Bazdyrev, Victoria Karetnikova, Olga Barbarash

**Affiliations:** Federal State Budgetary Institution “Research Institute for Complex Issues of Cardiovascular Diseases”, Kemerovo 650002, Russia

**Keywords:** prefrailty, frailty syndrome, coronary artery disease, percutaneous coronary intervention, biological markers

## Abstract

Background: The aim of this study was to analyze the prevalence of prefrailty and frailty syndrome (FS) in patients with coronary artery disease (CAD), and the clinical and biological characteristics of frail patients undergoing elective percutaneous coronary intervention (PCI). Material and Methods: The study included 78 patients with CAD who were admitted to the clinic to undergo PCI. To detect prefrailty and FS in patients, we used a short physical performance test battery (10–12 points—no FS, 8–9 points—prefrailty, 7 or fewer points—FS). We used the RayBio^®^ Human ELISA Kit (Norcross, GA, USA), a highly sensitive and highly specific enzyme-linked immunosorbent assay, to determine the concentration of biological markers of inflammation (IL-6, IL-10, IL-13, IL-15, TNF-α) and bone, muscle, and fat remodeling (leptin, calcitonin, osteoprotegerin, osteocalcin, myostatin) in the serum of patients with coronary artery disease before planned PCI. Results: Taking into account the test battery score, the prevalence of FS in patients with CAD before elective PCI was 24.4%, the prevalence of prefrailty was 33.3%. According to the results of the study, older women with type 2 diabetes in their history were significantly more likely to be frail. Studying a wide range of biological markers of inflammation and musculoskeletal and fat remodeling, we noted lower levels of calcitonin (2.60 [1.50; 5.85] pg/mL, *p* = 0.018) and osteoprotegerin (0.80 [0.60; 1.20] ng/mL, *p* = 0.025) in the serum of frail patients with CAD. Later we confirmed the results by correlation analysis. Moreover, we found an association between FS and higher serum leptin levels in patients with CAD before elective PCI. Conclusion: The results of the study confirm the high prevalence of prefrailty (33.3%) and FS (24.4%) in patients with CAD. Older women with type 2 diabetes in their history were significantly more likely to be frail. At the same time, the presence of FS is associated with lower levels of calcitonin and osteoprotegerin, and higher levels of leptin in the serum of frail patients before elective PCI.

## 1. Introduction

The prevalence of coronary artery disease (CAD) is increasing with the average life expectancy of the world’s population. Choosing an appropriate myocardial revascularization strategy for elderly patients is quite difficult due to the high level of comorbidity in this cohort of patients [1]. A number of studies have indicated that elderly patients with CAD have a more favorable prognosis after percutaneous coronary intervention (PCI) [2,3]. However, there were no statistically significant differences in cumulative 10-year mortality from all causes in a meta-analysis of randomized controlled trials comparing PCI using drug-coated stents and coronary artery bypass grafting in patients with left coronary artery trunk lesions [4]. One of the understudied but important adverse factors is frailty syndrome (FS), a complex condition in which the physiological reserves of the body decrease. FS in the elderly is characterized by its multidimensionality, involving the decline of physical, biological, social, and psychological domains, which damage homeostatic reserves and, therefore, increase vulnerability to stressors [5]. According to various studies, the prevalence of frailty in the elderly population ranges from 4.0% to 59.1% [6]. Such heterogeneous data are caused by the lack of the “gold” standard for the diagnosis of FS, and the gender, age, and nosological characteristics of the studied samples.

According to the results of a number of studies, FS can be an independent predictor of an unfavorable prognosis in patients undergoing PCI, both in short- and long-term follow-up [7,8,9,10]. Nevertheless, there are very few works devoted to the study of the role of FS in patients undergoing PCI. It is necessary to take into account the importance of cardiovascular diseases (CVDs), particularly CAD, in affecting the prevalence of FS. On the other hand, frail patients tend to be more burdened with CVD than their healthy peers. The pathophysiological features of the development of FS remain poorly understood. Despite the fact that frailty and atherosclerosis possibly share common pathogenetic mechanisms, there are no data on the key biological markers actively involved in the development of FS in CAD patients. One of the most popular theories of aging is based on the work of French scientists C. Franceschi et al. [11]. According to their theory, aging is associated with immune dysregulation, characterized by high levels of circulating inflammatory biomarkers such as interleukin 1 (IL-1), IL-8, IL-10, IL-13, C-reactive protein, and tumor necrosis factor alpha (TNF-α), even in the absence of risk factors or clinical manifestations [12,13]. Such indicators as myostatin and cytokines (for example, IL-6, IL-15, TNF-α), collectively referred to as myokines, can reflect muscle dysfunction in the case of FS. They are released from skeletal myocytes and affect muscles and bones. At the same time, there are few studies on the role of myokines in patients with FS and severe coronary lesions. Similarly, osteokines, including osteocalcin, osteoprotegerin, and bone matrix proteins, are released from bone cells and participate in the regulation of musculoskeletal homeostasis [14]. Adipokines actively involved in the regulation of the musculoskeletal system include TNF-α, IL-6, adiponectin, IL-13, IL-15, leptin, myostatin, sclerostin, osteopontin, and calcitonin. 

Thus, there are very few studies examining the prevalence of FS, its pathophysiological features of development, and the role of FS in CAD patients undergoing PCI. Nevertheless, frailty is seen as an unfavorable prognostic factor for patients undergoing various invasive treatment strategies. These facts served as a prerequisite for conducting this study aimed at assessing the prevalence of prefrailty and FS, describing the portrait of a frail patient, as well as studying the role of proinflammatory biological markers and markers of musculoskeletal and fat remodeling in the development of FS in CAD patients before routine PCI. 

## 2. Materials and Methods

The registry-based observational study conducted from 2023 to 2024 at the Cardiology Department of the Federal State Budgetary Institution “Research Institute for Complex Issues of Cardiovascular Diseases” (Kemerovo) included 78 patients with CAD who were scheduled for routine PCI. Before the inclusion in the study, patients signed an informed consent approved by the Institutional Review Board of the Federal State Budgetary Institution “Research Institute for Complex Issues of Cardiovascular Diseases”, Protocol No. 12 dated 27 December 2019. CAD diagnosis was verified on the basis of the ESC-2019 recommendations for the diagnosis and treatment of chronic coronary syndromes (stable CAD), the presence of angina, anamnesis data, examination methods including electrocardiography, echocardiography, daily monitoring of electrocardiography, and coronary angiography. The functional class (FC) of angina was assessed according to the classification of the Canadian Cardiovascular Society (CCS, 1976). The FC of heart failure (HF) was evaluated according to the classification of the New York Heart Association (NYHA, 1964). 

Patients underwent a short physical performance battery test (SPPB), 10–12 points—no FS, 8–9 points—prefrailty, 7 or fewer points—FS, to screen for frailty and prefrailty before PCI [15].

The main inclusion criteria were as follows: age over 18 years; stable CAD at the time of hospitalization (angina pectoris I–III FC and/or postinfarction cardiosclerosis (PICS)); hospitalization due to planned PCI. The exclusion criteria were the following: neuromuscular diseases; chronic heart failure (CHF) IV FC; uncontrolled arterial hypertension (AH); a combination of CAD and valvular heart defects; the presence of severe concomitant diseases that worsen mental and somatic health; traumatic brain injuries; inability to understand and (or) follow the procedures of the research protocol; refusal (withdrawal of consent) to participate in the study. 

We used the RayBio^®^ Human ELISA Kit (Norcross, GA, USA), a highly sensitive and highly specific enzyme-linked immunosorbent assay, to determine the concentration of the biological markers of inflammation (IL-6, IL-10, IL-13, IL-15, TNF-α) and bone, muscle, and fat remodeling (leptin, calcitonin, osteoprotegerin, osteocalcin, myostatin) in the serum of patients with coronary artery disease before planned PCI. 

Statistical processing of the results was carried out using the IBM SPSS Statistics 26.0.0 software. Absolute and relative indicators (%) were used to describe qualitative variables. The normality of the distribution of quantitative variables was assessed using the Shapiro–Wilk test. Most quantitative features were non-normal (Shapiro–Wilk W test 0.7–0.9; *p* ≤ 0.05) and were represented by the median and interquartile range (Me [Q1; Q3]). Quantitative variables with normal distribution were presented as mean and standard deviation Me (SD). To assess the statistical significance of differences in qualitative variables for three independent groups, the Pearson chi-squared test with the Yates continuity correction was used. In cases where the number of expected observations in any of the cells of the four-cell table was less than 5, the exact Fisher test was used to assess the significance of the differences. The Kruskal–Wallis test with the Holm–Bonferroni correction or the Dunnett’s test were used to compare three independent groups on a quantitative basis. The direction and strength of the relationship between the two quantitative indicators were assessed using Spearman’s rank correlation (in the case of non-normal distribution). The dependence of the quantitative variable on the studied variables was calculated using the linear regression method. The differences were considered statistically significant at *p* ≤ 0.05. 

## 3. Results

The mean age of patients was 67.00 ± 7.31 years; the gender distribution was equal. A total of 75 (96.2%) patients had hypertension in their history, 44 (56.4%) patients had suffered a myocardial infarction, and 63 (80.8%) patients had HF with preserved left ventricular ejection fraction (LVEF). Single-, double- and triple-vessel diseases of the coronary arteries had the same prevalence (27 (34.6%), 27 (34.6%), 24 (30.8%), respectively). A total of 46 (59.0%) patients had undergone PCI in the past and 14 (17.9%) patients had atrial fibrillation (AF) in the preoperative period. In total, 27 (34.6%) patients had type 2 diabetes mellitus (DM) in their history, 25 (32.1%) patients had chronic kidney disease (CKD), and five (6.4%) patients had suffered stroke (Table 1).

According to the results of the SPPB, all patients were divided into three groups: patients without FS were included in the group *n*_0_ = 33 (42.3%); patients with prefrailty were included in the group *n*_1_ = 26 (33.3%), and patients with FS were included in the group *n*_2_ = 19 (24.4%). The groups significantly differed in age and gender (Table 2). The oldest patients were most likely to be included in the FS and prefrailty groups—68.63 ± 5.85 and 67.50 ± 5.19 years; the youngest patients were likely to be included in the group without FS—63.09 ± 8.72 years. Women were significantly more likely than men to present with manifestations of FS (69.2% of women had FS and 33.3% of women did not have FS, *p* = 0.019). The result of the comparative statistical analysis showed that patients with FS were more likely to have type II DM in the anamnesis (42.1% in the group with FS, 50.0% in the group with prefrailty, and 18.2% in the group without FS, *p* = 0.028, respectively), as well as higher body weight (30.36 [27.24; 36.59] kg/m^2^ and 28.01 [23.85; 30.19] kg/m^2^, *p* = 0.013, respectively). Comorbid diseases in the groups were comparable according to the rest of the anamnesis data.

Next, we performed an enzyme immunoassay to assess the concentration of biomarkers of inflammation and musculoskeletal and fat remodeling in the serum of patients with coronary artery disease before planned PCI (Table 3). The results revealed that patients had higher serum concentrations of leptin (18.15 [9.15; 53.50] ng/mL) and myostatin (19.95 [16.40; 23.60] ng/mL), as well as a tendency towards lower levels of calcitonin (3.40 [1.80; 6.00] pg/mL), osteoprotegerin (1.10 [0.80; 1.30] ng/mL), and osteocalcin (15.05 [11.45; 25.67] ng/mL) compared with the mean values of these indicators in healthy participants in previous studies. The serum concentration of inflammatory biomarkers in CAD patients did not exceed the upper limits of the norm (IL-6 (0.75 [0.40; 2.02] pg/mL), IL-10 (0.75 [0.30; 1.60] pg/mL), IL-13 (3.70 [2.10; 10.80] pg/mL), IL-15 (0.40 [0.20; 0.60] pg/mL), TNF-α (0.90 [0.55; 1.40] pg/mL)) [16,17,18,19,20,21,22,23].

SPPB tests showed similar levels of such proinflammatory biological markers as IL-6, IL-10, IL-13, IL-15, and TNF-α in CAD patients with FS. Calcitonin levels were significantly lower among CAD patients with FS (2.60 [1.50; 5.85] pg/mL, *p* = 0.018), as well as osteoprotegerin (0.80 [0.60; 1.20] pg/mL, *p* = 0.025). There were no significant differences in the serum levels of leptin, osteocalcin, and myostatin in frail patients with CAD.

Then, we conducted a correlation analysis of the relationship between the SPPB scores and the serum level of biomarkers of inflammation and musculoskeletal and fat remodeling in patients before elective PCI using Spearman’s rank correlation (Table 4). When assessing the relationship between the level of leptin in the serum of CAD patients and SPPB scores, we found a weak inverse correlation. Thus, with a 1-point increase in the SPPB score, one can expect a decrease in serum leptin levels by 5.078 ng/mL. When evaluating the association between the serum level of calcitonin in patients undergoing planned PCI and SPPB scores, we found a weak direct relationship. With a 1-point increase in the SPPB score, one can expect an increase in calcitonin by 0.723 pg/mL. When evaluating the association between the serum level of osteoprotegerin in the serum of CAD patients and SPPB scores, we found a moderate direct relationship. With a 1-point increase in the SPPB score, one can expect an increase in osteoprotegerin by 0.056 ng/mL.

Thus, prefrailty was found in 33.3% of CAD patients, and FS was found in 24.4% of patients using SPPB scores. According to the results of our study, older women with type 2 DM in their history were significantly more likely to have frailty. At the same time, serum levels of calcitonin and osteoprotegerin were significantly lower in frail CAD patients (2.60 [1.50; 5.85] pg/mL, *p* = 0.018; 0.80 [0.60; 1.20] ng/mL, *p* = 0.025, respectively) in terms of the entire spectrum of studied biological markers of inflammation and musculoskeletal and fat remodeling.

## 4. Discussion

Unfortunately, at the moment, frail patients with CAD remain the least studied in terms of using various methods of myocardial revascularization. This may be due to the fact that elderly patients are more often excluded from large clinical trials on cardiovascular interventions due to the high risk of complications and limited life expectancy. Moreover, the lack of a “gold” standard for the diagnosis of FS affects the prevalence of frailty in patients undergoing PCI. Thus, according to the results of our study, 24.4% of patients undergoing PCI can be diagnosed with frailty on the basis of the SPPB. A study using the Clinical Frailty Scale (CFS, Canadian Study of Health and Aging Clinical Frailty Scale) identified 15.9% of patients over 65 years of age before PCI as frail [10]. In one of the large meta-analyses of studies devoted to the role of FS in CAD patients after PCI, the prevalence of FS ranged from 11.0% to 28.1%; the authors of those studies used the CFS scale and Fried frailty phenotype, respectively [24]. In a study by S. Qayyum et al. [25], FS was detected in 28.0% of patients according to the Fried phenotype and 26.1% when using the Edmonton Frail Scale (EFS). Thus, the prevalence of FS in patients with PCI remains quite high despite the use of different diagnostic approaches. 

Moreover, frail patients undergoing PCI are likely to have multiple comorbidities. According to the results of a study, in the case of no restrictions on the lower age limit of patients (18 years and older), older women with type II DM in their history were significantly more likely to be frail. Famously, males and females exhibit distinct risk profiles of CAD. Although both sexes share common risk factors such as DM, AH, and dyslipidemia, their influence on the development and progression of CAD differs substantially. Other nontraditional risk factors like psychosocial stress and systemic inflammation have been reported to have a more profound impact on women, acting as indirect risk enhancers for the development and progression of CAD. One such risk factor for women may be FS. The study by H. Shimono et al. [10] did not reveal any significant differences in gender and the prevalence of coronary risk factors such as AH, DM, dyslipidemia, and smoking; however, stroke in the history, peripheral artery disease, and hemodialysis were significantly more common in frail CAD patients. In a similar study by K. Kanenawa et al. [26], frail patients were older and had more concomitant diseases such as CKD and anemia. Nevertheless, a number of other studies found no significant differences in the clinical and anamnestic characteristics of CAD patients before PCI [7,27]. 

The number of studies on the pathophysiological features of the development of FS in patients with various CVD is extremely low. The relationship established in this study between FS and higher serum leptin levels in CAD patients requires further study, taking into account a number of limitations and the insufficient number of data in this field of study as a whole. High levels of leptin through the activation of proinflammatory cytokines can lead to muscle atrophy [28]. In the study by K. Kohara et al. [29], leptin levels were higher in the serum of elderly patients with sarcopenia and sarcopenic obesity compared with healthy elderly participants. In another study, frail elderly patients with manifestations of cachexia had significantly lower serum levels of leptin [30]. Thus, the study of the role of leptin in the development of FS requires additional research. The number of studies on the role of calcitonin and osteoprotogerin levels, which are actively involved in maintaining the strength of bones, in the serum of frail patients is limited. However, several studies have revealed associations between low calcitonin levels and FS in elderly patients with various comorbid pathologies, similarly to our study [31,32]. In the work by A. Valentini et al. [33], osteoprotogerin was also considered as a marker of FS; scientists hypothesized that a marked increase in the level of osteoprotegerin in the serum of elderly patients may reflect the progressive accumulation of damage in organs leading to the development of severe FS. But in our study, the level of this biological marker was significantly higher in patients without FS, which also requires additional study.

## 5. Conclusions

The results of this study confirm that the prevalence of prefrailty and FS in CAD patients before elective PCI is extremely high, which may be one of the most important factors affecting the prognosis of this cohort of patients. Therefore, further research is needed for a more detailed consideration and study of the role of FS and the features of atherosclerosis and pathological aging of the body in patients undergoing PCI.

## Figures and Tables

**Table 1:** Baseline clinical and anamnestic characteristics of patients with stable coronary artery disease.

Parameter	Patient Characteristics *(n =* 78)
Mean age, years, Me ± SD	67.00 ± 7.31
Men, *n* (%)	39 (50.0)
BMI, kg/m^2^, Me [Q1; Q3]	28.81 [24.98; 32.55]
Smoking/stopped smoking less than 3 months ago, *n* (%)	11 (14.1)
PICS, *n* (%)	44 (56.4)
CHF II–III FC, *n* (%)	71 (91.1)
HF with preserved LVEF, *n* (%)	63 (80.8)
PCI in history, *n* (%)	46 (59.0)
Single-vessel disease, *n* (%)	27 (34.6)
Double-vessel disease, *n* (%)	27 (34.6)
Triple-vessel disease, *n* (%)	24 (30.8)
Stroke in history, *n* (%)	5 (6.4)
AH, *n* (%)	75 (96.2)
AF in preoperative period, *n* (%)	14 (17.9)
Type 2 DM, *n* (%)	27 (34.6)
COPD, *n* (%)	1 (1.3)
CKD, *n* (%)	25 (32.1)

Note: BMI—body mass index, PICS—postinfarction cardiosclerosis, CHF—chronic heart failure, HF—heart failure, LVEF—left ventricular ejection fraction, PCI—percutaneous coronary intervention, AH—arterial hypertension, AF—atrial fibrillation, DM—diabetes mellitus, COPD—chronic obstructive pulmonary disease, CKD—chronic kidney disease, FC—functional class.

**Table 2:** Clinical and anamnestic characteristics of patients before elective percutaneous coronary intervention, depending on the presence of prefrailty and frailty syndrome.

Parameter	Group of Patients Without FS, *n*_0_ = 33 (42.3%)	Group of Patients with Prefrailty, *n*_1_ = 26 (33.3%)	Group of Patients with FS, *n*_2_ = 19 (24.4%)	*p*
Mean age, years, Me ± SD	63.09 ± 8.72	67.50 ± 5.19	68.63 ± 5.85	0.022 * p_w/o FS − P/F_ = 0.050 * p_w/o FS − FS_ = 0.023 *
Men, *n* (%)	22 (66.7)	8 (30.8)	9 (47.4)	0.023 * p_w/o FS − P/F_ = 0.019 *
BMI, kg/m^2^, Me [Q1; Q3]	28.01 [23.85; 30.19]	30.38 [25.72; 32.43]	30.36 [27.24; 36.59]	0.044 * p_w/o FS − FS_ = 0.013 *
Smoking/stopped smoking less than 3 months ago, *n* (%)	5 (15.2)	3 (11.5)	3 (15.8)	0.898
PICS, *n* (%)	17 (51.5)	18 (69.2)	9 (47.4)	0.260
CHF class II–III, *n* (%)	27 (81.8)	25 (96.2)	19 (100.0)	0.058
HF with preserved LVEF, *n* (%)	24 (72.7)	21 (80.8)	18 (94.7)	0.536
PCI in history, *n* (%)	22 (66.7)	13 (50.0)	11 (57.9)	0.373
Single-vessel disease, *n* (%)	14 (42.4)	8 (30.8)	5 (26.3)	0.750
Double-vessel disease, *n* (%)	11 (33.3)	9 (34.6)	7 (36.8)	
Triple-vessel disease, *n* (%)	8 (24.2)	9 (34.6)	7 (36.8)	
Stroke in history, *n* (%)	2 (6.1)	1 (3.8)	2 (10.5)	0.661
AH, *n* (%)	31 (93.9)	25 (96.2)	19 (100.0)	0.549
AF in preoperative period, *n* (%)	5 (15.2)	5 (19.2)	4 (21.1)	0.848
Type 2 DM, *n* (%)	6 (18.2)	13 (50.0)	8 (42.1)	0.028 * p_w/o FS − P/F_ = 0.028 *
COPD, *n* (%)	0 (0.0)	0 (0.0)	1 (5.3)	0.207
CKD, *n* (%)	11 (33.3)	8 (30.8)	6 (31.6)	0.977

Note: * Statistically significant differences (*p* ≤ 0.050).

**Table 3:** Biomarkers of inflammation and musculoskeletal and fat remodeling in the serum of patients before elective percutaneous coronary intervention, depending on the presence of prefrailty and frailty syndrome.

Parameter	Type of Patients	Me	Q1; Q3	*p*
IL-6, pg/mL	Without FS	0.90	0.40; 1.95	0.372
	With prefrailty	0.75	0.40; 1.67	
	FS	0.55	0.33; 0.93	
IL-10, pg/mL	Without FS	1.00	0.30; 1.60	0.785
	With prefrailty	1.05	0.40; 1.48	
	FS	0.60	0.20; 1.70	
IL-13, pg/mL	Without FS	6.00	2.00; 11.25	0.494
	With prefrailty	2.60	2.10; 5.30	
	FS	4.10	2.10; 6.60	
IL-15, pg/mL	Without FS	0.45	0.40; 0.57	0.105
	With prefrailty	0.15	0.10; 0.50	
	FS	0.20	0.18; 0.23	
TNF-α, pg/mL	Without FS	0.90	0.65; 1.30	0.876
	With prefrailty	1.05	0.55; 1.38	
	FS	0.95	0.55; 1.55	
Leptin, ng/mL	Without FS	13.10	5.90; 37.50	0.141
	With prefrailty	24.55	14.72; 57.50	
	FS	40.00	8.60; 68.05	
Calcitonin, pg/mL	Without FS	4.30	2.80; 6.75	0.017 * p_P/F − w/o FS_ = 0.018 *
	With prefrailty	2.10	1.20; 4.20	
	FS	2.60	1.50; 5.85	
Osteoprotegerin, ng/mL	Without FS	1.20	0.80; 1.60	0.018 * p_FS − w/o FS_ = 0.025 *
	With prefrailty	1.00	0.80; 1.18	
	FS	0.80	0.60; 1.20	
Osteocalcin, ng/mL	Without FS	15.80	9.40; 26.40	0.752
	With prefrailty	14.20	10.47; 28.52	
	FS	17.30	13.50; 23.40	
Myostatin, ng/mL	Without FS	20.80	18.20; 24.00	0.376
	With prefrailty	19.50	15.72; 23.10	
	FS	19.40	15.85; 22.25	

Note: * Statistically significant differences (*p* ≤ 0.050).

**Table 4:** Biomarkers of inflammation and musculoskeletal and fat remodeling in the serum of patients depending on the presence of prefrailty and frailty syndrome.

Parameter	ρ	Strength of Association According to Chaddock Scale	*p*
IL-6, pg/mL	0.187	weak	0.180
IL-10, pg/mL	0.139	weak	0.259
IL-13, pg/mL	0.103	weak	0.384
IL-15, pg/mL	0.383	moderate	0.064
TNF-α, pg/mL	−0.156	weak	0.284
Leptin, ng/mL	−0.251	weak	0.027 *
Calcitonin, pg/mL	0.246	weak	0.034 *
Osteoprotegerin, ng/mL	0.316	moderate	0.005 *
Osteocalcin, ng/mL	−0.112	weak	0.330
Myostatin, ng/mL	0.181	weak	0.113

Note: * Statistically significant differences (*p* ≤ 0.050).

## Data Availability

The original contributions presented in the study are included in the article, further inquiries can be directed to the corresponding author.

## Abbreviations

AH	arterial hypertension
CAD	coronary artery disease
IL	interleukin
BMI	body mass index
SPPB	short physical performance battery
LVEF	left ventricular ejection fraction
PICS	postinfarction cardiosclerosis
DM	diabetes mellitus
HF	heart failure
FS	frailty syndrome
CVD	cardiovascular diseases
FC	functional class
TNF-α	tumor necrosis factor alpha
AF	atrial fibrillation
CKD	chronic kidney disease
COPD	chronic obstructive pulmonary disease
CHF	chronic heart failure
PCI	percutaneous coronary intervention
